# Relationship Between Oral Intake and Sarcopenia in Patients with Disease-Related Malnutrition †

**DOI:** 10.3390/nu17132129

**Published:** 2025-06-27

**Authors:** Paloma Pérez-López, Juan José López-Gómez, Olatz Izaola-Jauregui, Jaime González-Gutiérrez, Lucía Estévez-Asensio, Isabel Pérez-Mellen, Eva López-Andrés, David Primo-Martín, Esther Delgado-García, Rebeca Jiménez-Sahagún, Beatriz Ramos-Bachiller, Daniel Antonio de Luis-Román

**Affiliations:** 1 Hospital Clínico Universitario de Valladolid, 47003 Valladolid, Spaindprimoma@saludcastillayleon.es (D.P.-M.); dadluis@yahoo.es (D.A.d.L.-R.); 2 Centro de Investigación en Endocrinología y Nutrición (IENVA), Universidad de Valladolid, 47002 Valladolid, Spain

**Keywords:** sarcopenia, disease-related malnutrition, oral intake, protein intake

## Abstract

**Background/Objectives**: Assessing oral intake in patients with disease-related malnutrition (DRM) and sarcopenia remains a clinical challenge. This study aimed to evaluate the relationship between oral intake adjusted to nutritional requirements and the presence of sarcopenia in patients with DRM. **Methods:** This was a prospective observational study involving 118 outpatients with DRM, diagnosed according to Global Leadership Initiative on Malnutrition criteria. Sarcopenia was assessed using the European Working Group on Sarcopenia in Older People criteria. A 3-day dietary intake record was collected at the beginning of nutritional follow-up. Caloric (kcal/day) and protein (g/day) intakes were calculated. Energy needs were estimated using the Harris-Benedict equation with stress factors, and protein needs were set at 1.5 g/kg/day. Intake was categorized based on whether energy and protein intake exceeded or fell below 70% of requirements. **Results:** The mean age was 62.2 years, and 58.8% were female. Sarcopenia was present in 42% of patients. No significant difference was found in body mass index between patients with and without sarcopenia. Mean caloric intake was 29.6 kcal/kg/day and protein intake was 1.3 g/kg/day. Average fulfilment was 78.3% for energy and 86.8% for protein. Patients with sarcopenia had significantly lower intake of calories and macronutrients. Sarcopenia was more prevalent in those with <70% fulfilment of caloric and protein requirements. Multivariate analysis showed increased risk of sarcopenia (Odds ratio (OR): 4.27; 95% Confidence Interval (CI): 1.30–14.03; *p* = 0.017) and severe malnutrition (OR: 5.17; 95% CI: 1.63–16.42; *p* < 0.01) in patients with low protein intake. **Conclusions:** In patients with DRM, insufficient intake of calories and protein was associated with a higher prevalence of sarcopenia. There was an increased risk of sarcopenia and severe malnutrition in patients with lower protein intake.

## 1. Introduction

Disease-related malnutrition (DRM) is a highly relevant pathology that poses a challenge to our healthcare system. It is estimated that DRM has a prevalence between 5 and 15% of the outpatient population, between 20% and 50% in hospitalized patients and up to 60% in institutionalized patients [1,2]. Its diagnosis is made through the Global Leadership Initiative on Malnutrition (GLIM) criteria [3], requiring at least one phenotypic criterion, related to anthropometry and/or body composition, and one etiological criterion for its definition, associated with the cause of malnutrition (inflammatory load or decreased oral intake) [3].

DRM can be related to another pathology of high prevalence, such as sarcopenia. Sarcopenia is defined as a progressive and generalized disorder associated with a reduction in the quantity and quality of muscle, as well as its function, according to the criteria of the European Working Group on Sarcopenia in Older People (EWGSOP2) [4]. This reduction leads to increased falls, fractures and physical disability, decreased function, loss of quality of life and increased mortality [5,6,7]. In Spain, it is coded as M62.84 under the ICD-10-ES system, and it affects up to 33% of older women and 10% of older men in urban settings, and up to 37% of elderly residents in nursing homes of this country [8]. Across Europe, the prevalence ranges from 11% to 20% among adults over 65, with projections reaching 22% by 2045 [9].

The onset of sarcopenia is influenced by multiple factors, due to the imbalance between protein anabolic and catabolic processes. The European Society for Clinical Nutrition and Metabolism (ESPEN) recommends initiating medical nutritional therapy in patients with DRM and sarcopenia [10,11], and a large part of the clinical trials have been focused on increasing the protein load in these patients, as well as interventions on physical activity [12,13]. However, the assessment of oral intake, and especially protein intake, remains a challenge at present, due in part to the absence of standardized tests for its assessment and the need to use a large number of resources and time to categorize and quantify it.

In addition to the assessment of dietary intake, for an adequate categorization of malnutrition and sarcopenia we need to assess body composition, muscle strength and function and biochemical parameters [14,15]. In this global approach, the assessment of muscle mass, with emerging techniques such as Bioelectrical Impedance Analysis (BIA) and muscle ultrasound plays an essential role [16,17,18,19].

In view of the above-mentioned, the aim of this study was to evaluate the relationship between oral intake adjusted to nutritional requirements with the presence of sarcopenia in patients with DRM.

## 2. Materials and Methods

### 2.1. Type of Study

A cross-sectional study was developed in 118 outpatients diagnosed with DRM by GLIM criteria [3]. Patients were recruited at the Clinical Nutrition Unit of the Hospital Clínico Universitario of Valladolid (Spain) between January 2021 and December 2023. After signing the informed consent, clinical and nutritional data were collected. Anthropometry, BIA, manual grip strength, and muscle mass and quality were assessed by muscle ultrasound of rectus femoris (RF). The study was approved by the ethics committee of the Valladolid East Area with code PI 22-907 on 13 October 2022; and was performed following the principles of the Declaration of Helsinki.

### 2.2. Study Population

Selected patients met the following inclusion criteria: outpatients diagnosed with DRM by GLIM criteria and age over 18 years [20]. Exclusion criteria were: uncontrolled liver disease, chronic kidney disease above stage IV, and patients who did not sign the informed consent form.

### 2.3. Studied Parameters

Clinical variables: diagnosis of malnutrition and sarcopenia: Severe malnutrition was considered to be those patients with phenotypic GLIM criteria of more than 10% weight loss in the last six months or >20% in the last year; or a body mass index (BMI) < 18.5 kg/m^2^ in those younger than 70 years or <20 kg/m^2^ in those older than 70 years [3]. To determine the diagnosis of sarcopenia, the EWGSOP2 criteria were used [4]. Low muscle strength (or dynapenia) was considered a handgrip strength less than 16 kg in women and less than 27 kg in men.

Anthropometric variables: Body height (meters) was measured using a calibrated height measurement scale (Omron, Los Angeles, CA, USA). Body weight (kg) was measured while the subjects were minimally clothed and not wearing shoes, using digital scales (Omron). BMI was calculated using the formula: weight in kg divided by height in m^2^.

Dietary intake: Over the course of a 72-h period, all patients meticulously documented their dietary intake to facilitate an accurate estimation of their average daily consumption of calories and macronutrients. The dietary records encompassed a total of three days—two weekdays and one weekend day—to provide a representative snapshot of typical eating patterns. These collected records were subsequently analysed using specialized nutritional assessment software (Dietsource^®^, Version 3.0, Nestlé, Geneva, Switzerland), ensuring precise and standardized evaluation of nutrient intake.

Caloric (kcal/day) and protein intake (grams/day) were calculated. Energy requirements (Harris-Benedict equation × stress factor) and protein requirements (1.5 g/kg/day) were evaluated [21] Resting Metabolic Rate (RMR) for males: (9.65 × weight in kg) + (573 × height in m) − (5.08 × age in years) + 260; RMR for females: (7.38 × weight in kg) + (607 × height in m) − (2.31 × age in years) + 43. Fulfilment of caloric and protein requirements was evaluated. The results were stratified according to caloric and protein intake greater (CAL > 70 and PROT > 70) or less (CAL < 70 and PROT < 70) than 70% of calculated requirements.

Body composition:

- BIA: (BIA NutriLab^®^; EFG Akern, Akern, Pisa, Italy) was performed between 8:00 and 9:15, after an overnight fast and after a time of 15 min in supine position. Resistance (R) and reactance (Xc) parameters were measured. The phase angle (PA) was calculated with: PA = ((Xc/R) × 180°/π) [22].
- Muscle ultrasound of the RF of the dominant lower extremity was performed with a 10 to 12 MHz probe and a multifrequency linear array (Mindray Z60, Mindray Medical España S.L., Madrid, Spain). The measurement was performed with the patient in supine decubitus, without compression, at the level of the lower third from the superior pole of the patella and the anterosuperior iliac spine [15]. The measured parameters to assess muscle mass were anteroposterior (Y-axis) and transverse (X-axis) muscle thickness (cm), muscle area (RFMA, cm^2^). The variable used to assess muscle quality was the X-Y index ((X-axis/Y-axis)/height^2^) which relates transverse and anteroposterior muscle thickness.

Muscle function: Muscle function was obtained with handgrip strength (JAMAR^®^ dynamometer, Preston, Jackson, MO, USA). The measurement was taken with the patient seated with the dominant arm in right angle position. Three determinations were made and the highest value of the three was chosen.

### 2.4. Statistical Analysis

The database was registered with permission from the National Data Protection Agency. The data collected were stored in a database using the SPSS 23.0 statistical program (SPSS Inc., Chicago, IL, USA). Continuous variables were presented as mean and standard deviation, while parametric variables were analyzed using Student’s *t*-test for unpaired samples. For nonparametric variables, Friedman, Wilcoxon and Mann-Whitney U tests were used. To compare variables in more than two groups, the ANOVA U test was applied with the Bonferroni post hoc test. The analysis of variables at different times of the study was performed by multivariate analysis of variance. Qualitative variables were expressed as percentages and analyzed using the Chi-square test, with Fisher and Yates adjustments when necessary. A multivariate analysis was performed using binary logistic regression to assess risk factors for sarcopenia. The risk was calculated through estimation of an odds ratio (OR) with 95% confidence intervals (CI). A *p* value of less than 0.05 was considered statistically significant.

## 3. Results

### 3.1. Descriptive Analysis

**Figure 1:**
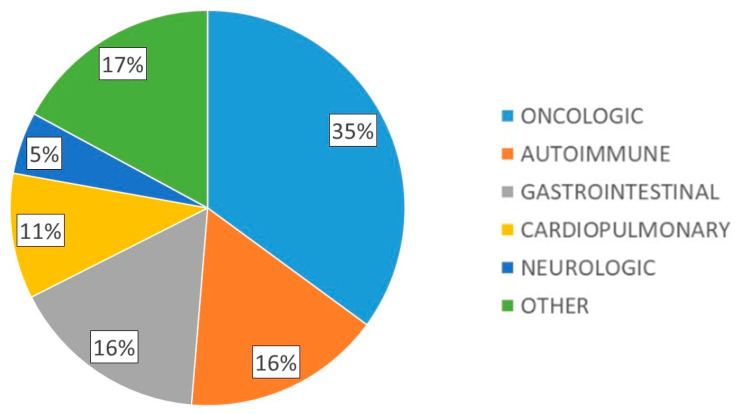
Main underlying pathologies of the patients studied.

A total of 118 patients with DRM were included, 56.9% with severe malnutrition and 42% with sarcopenia. Mean age was 62.2 (17.0) years, 58.5% were female, with mean body weight 55.0 (11.9) kg, mean BMI 21.5 (3.9) kg/m^2^, dominant hand dynamometry 21.1 (8.1) kg, BIA parameters: R 596.9 (105.6) ohms, Xc 51.0 (10.8) ohms, PA 4.9 (0.8)° and muscle ultrasound parameters: X-axis 3.34 (0.7) cm, Y-axis 1.1 (0.5) cm, X-Y index 0.4 (0.5), RFMA 3.1 (1.1) cm^2^. The main underlying pathologies of the patients studied are shown in Figure 1.

When comparing patients with and without sarcopenia, statistically significant differences were observed in age, weight, handgrip strength, Xc, PA and RFMA [Table 1].

**Figure 2:**
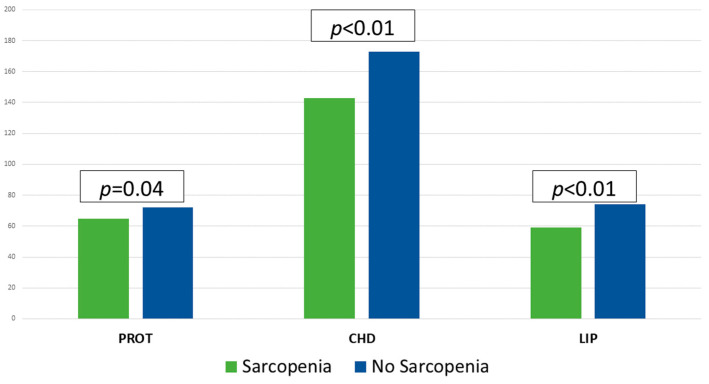
Macronutrient intakes (g/day) in patients with and without sarcopenia using Student’s *t*-test. CHD: carbohydrates; LIP: lipids; PROT: proteins.

The mean caloric intake was 29.56 (10.26) kcal/day and protein intake was 1.30 g/kg/day. Macronutrient intake is shown in Figure 2. The fulfilment of requirements was 78.27% in calories and 86.77% in protein. 60.5% of the patients consumed less than 70% of the caloric requirements (CAL < 70) and 63.9% less than 70% of the protein requirements (PROT < 70). In those patients with sarcopenia (S) a lower caloric (S: 1394 (404) kcal/day; NonS: 1654 (432) kcal/day; *p* < 0.01) and macronutrient intake (Protein: S: 64.70 (19.63) g/day; NonS: 72.12 (18.23) g/day; *p* = 0.04), (Carbohydrates: S: 143.47 (52.66) g/day; NonS: 173.70 (51.64) g/day; *p* < 0.01), (Fats: S: 59.46 (18.95) g/day; NonS: 73.76 (23.11) g/day; *p* < 0.01), was observed [Figure 2].

### 3.2. Comparison Between Caloric and Protein Intake Higher and Lower than 70% for Nutritional Parameters

Table 2 shows the comparison between patients with caloric intake higher and lower than 70% for nutritional parameters. This comparison showed differences only in R, which was higher in those patients with lower caloric intake (618.8 (108.1) vs. 564.1 (93.7) kcal/day; *p* < 0.01). Regarding protein requirements, no differences in nutritional parameters were observed between patients with higher and lower than 70% oral intake (Table 3). A higher percentage of sarcopenia was observed in those patients with worse fulfilment of caloric (CAL < 70: 57.8%; CAL > 70: 43.7%; *p* = 0.02) and protein (PROT < 70: 48.8%; PROT > 70: 42.7%; *p* = 0.02) requirements.

### 3.3. Correlation Between Caloric Intake and Nutritional Parameters

When analyzing the correlation between caloric intake and nutritional parameters, direct moderate correlations were objectified with R (r: 0.385: *p* < 0.01) and Xc (r: 0.327: *p* < 0.01), and inverse with BMI (r: −0.228: *p* < 0.01) (Table 4).

### 3.4. Assessment of Risk Factors for Sarcopenia

When multivariate analysis was performed using binary logistic regression, there was an increased risk of sarcopenia in those patients who consumed less than 70% of protein requirements adjusting for age, sex and caloric intake (OR [CI]: 4.273 [1.302–14.028]; *p* = 0.017) (Table 5). No association with malnutrition was observed, but there was an increased risk of severe malnutrition in those with PROT < 70 (OR [CI]: 5.170 [1.628–16.416]; *p* < 0.01).

## 4. Discussion

Our findings show that in patients suffering from DRM, those diagnosed with sarcopenia had lower calorie and macronutrient intake compared to those without sarcopenia. In addition, patients with sarcopenia were generally older and had reduced body weight, muscle strength, reactance, phase angle and fat mass compared to those without sarcopenia. Moreover, sarcopenia was found to be more common in those who did not adequately meet their calorie and protein requirements, and caloric intake was also found to be directly related to certain parameters of bioelectrical impedance analysis, such as resistance and reactance. Finally, an increased risk of developing sarcopenia was identified in patients under 70% of dietary protein requirements.

Despite the importance of oral intake in the patient with DRM and sarcopenia, its evaluation continues to be a challenge to this day. Therefore, tools such as intake records and structured nutritional tests combining different parameters are an essential part of nutritional evaluation. This assessment of intake, added to other aspects of nutritional evaluation, highlighting the evaluation of muscle mass by BIA or muscle ultrasound of RF, has allowed us to achieve a more holistic assessment of the nutritional status of our patients [14,18].

Regarding the parameters of anthropometry, hand grip strength, BIA and muscle ultrasound of RF, the results of this study are in line with others previously published on malnutrition and sarcopenia [16,19,23]. When differentiating patients with and without sarcopenia, it was observed that the former presented a higher body weight, with no differences for BMI. This finding can be explained by the fact that BMI is not an adequate index to assess functionality (an essential part in the definition of sarcopenia) and this discordance highlights the need to use new parameters to assess body composition. These include BIA, where those patients with sarcopenia showed lower Xc and PA than those without sarcopenia. Both parameters, especially the PA, have been associated in numerous studies with mortality in patients with DRM [19,22].

Concerning oral intake in patients with sarcopenia, some studies show a decrease of up to 25% between the ages of 40 and 70, leading to insufficient intake in relation to energy and protein requirements and in parallel to a decrease in muscle mass, which leads to sarcopenia [24,25]. For instance, a meta-analysis using data of 8107 communitydwelling older adults from the PRevention Of Malnutrition In Senior Subjects in the EU (PROMISS) project showed that the prevalence of protein intake below 0.8, 1.0, and 1.2 g/kg/d was 29.1%, 54.3%, and 75.7%, respectively [26].

Regarding the assessment of oral intake, around 60% of patients with DRM were below 70% of their caloric and protein requirements, a significant figure compared to similar studies [12]. As shown in our study, there were hardly any significant differences between patients consuming more or less than 70% of their caloric and protein requirements, with disparate results in terms of hand grip strength, BIA and muscle ultrasound. Admittedly, those patients with lower adjustment to calorie-protein requirements had a higher prevalence of sarcopenia, with no differences in BIA, ultrasound or handgrip strength. This discordance could be because those patients with a diet better adjusted to their requirements (CAL > 70 and PROT > 70) could be those with worse nutritional parameters by initially who had already started nutritional treatment, that is, in the process of recovery.

On the other hand, those patients with sarcopenia and DRM showed a lower caloric intake, at the expense of the three types of macronutrients, which is congruent at a nutritional level, since a higher protein intake (mainly animal) usually leads to a higher fat intake, which in turn leads to a higher caloric count [10,11]. This highlights the need to establish treatments that increase protein intake in subjects with DRM, to avoid perpetuating this vicious circle between loss of function, immobilization and low intakes leading to sarcopenia [4,8].

Considering the correlations between caloric intake and nutritional parameters, it was observed that higher caloric intake was directly related to higher levels of resistance and reactance and inversely with BMI (weakly with the latter), without significant correlations with handgrip strength or muscle ultrasound parameters. Therefore, BIA seems to be the body composition technique with the best relationship to assess the caloric intake of these patients [19,22], always taking into account the limited sample size of our work.

Interestingly, this study incorporates body composition techniques such as BIA and RF muscle ultrasound. Currently, some studies such as the one by Koh et al. [27] show the impact of surgical prehabilitation with beta-hydroxymethylbutyrate in sarcopenic elderly people, incorporating artificial intelligence-assisted muscle ultrasound for the assessment of intramuscular fat, allowing functional improvements to be observed in subjects who received the supplementation. On the other hand, PA was a differentiating parameter in our work, being higher in those subjects without sarcopenia Norman et al. [28] showed that PA is an independent predictor of muscle strength and physical performance, even beyond lean mass indices. Thus, its inclusion as a complementary tool in the assessment of sarcopenia allows for a more functional and prognostic approach, especially useful in clinical settings where rapid and non-invasive assessments are required.

This study has certain strengths and limitations. As limitations: the relatively small sample size compared to big data studies; however, it is a real cohort with a wide variety of underlying pathologies, which allows these results to be transferred to other studies or population groups. On the other hand, although we do not use standardized tests for the assessment of intake, such as the Mini Nutritional Assessment or the Subjective Global Assessment, we do perform a complete 3-day dietary record. Moreover, we have an exhaustive knowledge of nutritional variables, with the use of emerging techniques such as BIA and muscle ultrasound, as well as anthropometric parameters, and our results are in line with those previously published on dietary intake, sarcopenia and DRM.

Future lines of research include automatize the assessment of oral intake by means of new technological tools to speed up data collection and thus assess the adjustment to caloric and protein requirements of our patients more accessible. On the other hand, artificial intelligence tools could allow us to better integrate data on oral intake assessment, body composition and functionality in order to personalize the most appropriate nutritional treatment for each patient.

## 5. Conclusions

In patients with disease-related malnutrition, the group with a diagnosis of sarcopenia showed lower caloric and macronutrient intake than those patients without sarcopenia. Patients with sarcopenia presented older age and lower values of body weight, dynamometry, reactance, phase angle and RFMA than those without sarcopenia. A higher prevalence of sarcopenia was observed in those patients with worse fulfilment of caloric and protein requirements. Caloric intake was directly correlated with BIA parameters such as endurance and reactance. Finally, there was an increased risk of sarcopenia and severe malnutrition in patients with lower protein intake.

## Figures and Tables

**Table 1:** Nutritional assessment in patients with and without sarcopenia using Student’s *t*-test.

n = 118	SARCOPENIA	NO SARCOPENIA	*p*-Value
Patients	50 (42%)	68 (58%)	-
Gender % (M/F)	43.4/56.6	40/60	*p* = 0.71
Age (years)	70.4 (12.0)	55.6 (17.7)	*p* < 0.01
Diabetes mellitus	21 (39.6%)	15 (23.1%)	*p* > 0.05
Oncologic (%)	19 (35.8%)	22 (33.8%)	*p* = 0.26
Autoimmune (%)	9 (17.0%)	10 (15.4%)	*p* = 0.26
Gastrointestinal (%)	6 (11.3%)	13 (20.0%)	*p* = 0.26
Body weight (kg)	52.5 (10.2)	57.0 (12.8)	*p* = 0.04
BMI (kg/m^2^)	21.2 (21.2)	21.8 (21.8)	*p* = 0.44
Dynamometry (kg)	15.7 (6.1)	25.4 (6.8)	*p* < 0.01
Resistance (ohm)	606.8 (104.5)	589.0 (106.6)	*p* = 0.37
Reactance (ohm)	47.0 (9.0)	54.1 (11.2)	*p* < 0.01
Phase Angle (°)	4.5 (0.7)	5.3 (0.8)	*p* < 0.01
X-axis (cm)	3.4 (0.7)	3.3 (0.7)	*p* = 0.44
Y-axis (cm)	1.0 (0.5)	1.2 (0.6)	*p* = 0.24
Y/X index	0.3 (0.5)	0.4 (0.5)	*p* = 0.24
RFMA (cm^2^)	2.9 (1.2)	3.3 (1.0)	*p* = 0.04

BMI: Body Mass Index; F: Female; M: Male; RFMA: Rectus Femoris Muscle Area.

**Table 2:** Nutritional assessment in patients with dietary intakes divided in two groups by fulfillment of 70% of their energy requirements using Student’s *t*-test.

N = 114	CAL < 70	CAL > 70	*p*-Value
Patients	69	45	-
Gender % (M/F)	57.8/42.2	29.6/70.4	*p* = 0.28
Age (years)	65.91 (14.10)	59.94 (18.54)	*p* = 0.07
Body weight (kg)	57.36 (12.67)	53.43 (11.29)	*p* = 0.08
BMI (kg/m^2^)	22.27 (3.88)	21.08 (3.86)	*p* = 0.11
Dynamometry (kg)	20.8 (7.4)	21.4 (9.2)	*p* = 0.71
Resistance (ohm)	618.8 (108.1)	564.1 (93.7)	*p* < 0.01
Reactance (ohm)	52.4 (11.4)	48.8 (9.8)	*p* = 0.08
Phase Angle (°)	4.9 (0.9)	5.0 (0.8)	*p* = 0.50
X-axis (cm)	3.3 (0.6)	3.6 (0.9)	*p* = 0.74
Y-axis (cm)	1.0 (0.4)	1.2 (0.8)	*p* = 0.17
Y/X index	0.3 (0.3)	0.5 (0.8)	*p* = 0.22
RFMA (cm^2^)	3.0 (1.2)	3.2 (1.1)	*p* = 0.37
Sarcopenia (%)	48.8	42.7	*p* = 0.52

BMI: Body Mass Index; CAL < 70: Caloric intake less than 70% of requirements; CAL > 70: Caloric intake greater than 70% of requirements; F: Female; M: Male; RFMA: Rectus Femoris Muscle Area.

**Table 3:** Nutritional assessment in patients with dietary intakes divided in two groups by fulfillment of 70% of their protein requirements using Student’s *t*-test.

N = 106	PROT < 70	PROT > 70	*p*-Value
Patients	73	43	-
Age (years)	57.13 (20.52)	64.02 (15.30)	*p* = 0.05
Dynamometry (kg)	20.9 (7.3)	21.4 (9.4)	*p* = 0.79
Resistance (ohm)	608.9 (104.1)	576.3 (106.2)	*p* = 0.11
Reactance (ohm)	52.4 (10.9)	48.5 (10.5)	*p* = 0.06
Phase Angle (°)	5.0 (0.9)	4.8 (0.7)	*p* = 0.46
X-axis (cm)	3.3 (0.7)	3.3 (0.7)	*p* = 0.99
Y-axis (cm)	1.1 (0.6)	1.0 (0.4)	*p* = 0.15
Y/X index	0.4 (0.6)	0.3 (0.4)	*p* = 0.40
RFMA (cm^2^)	3.2 (1.2)	2.9 (1.0)	*p* = 0.33
Sarcopenia (%)	54.8	41.4	*p* = 0.19

PROT < 70: Protein intake less than 70% of requirements; PROT > 70: Protein intake greater than 70% of requirements; RFMA: Rectus Femoris Muscle Area.

**Table 4:** Pearson’s correlation between caloric intake and nutritional parameters.

	N = 118	*p*-Value
BMI (kg/m^2^)	−0.228	*p* < 0.01
Dynamometry (kg)	−0.041	*p* = 0.09
Resistance (ohm)	0.385	*p* < 0.01
Reactance (ohm)	0.327	*p* < 0.01
Phase Angle (º)	0.017	*p* = 0.65
X-axis (cm)	−0.028	*p* = 0.43
Y-axis (cm)	−0.005	*p* = 0.82
Y/X index	−0.060	*p* = 0.60
RFMA (cm^2^)	0.019	*p* = 0.13

BMI: Body Mass Index; RFMA: Rectus Femoris Muscle Area.

**Table 5:** Multivariate analysis using binary logistic regression to identify risk factors of sarcopenia. CI: Confidence interval; F: Female; M: Male; OR: Odds ratio.

	OR	IC 95%	*p*-Value
Age (years)	1.07	1.04–1.11	<0.01
Gender (M/F)	0.48	0.55–3.55	0.48
Protein Intake (<70%)	4.27	1.30–14.03	0.017
Caloric intake (<70%)	1.33	0.53–3.31	0.77

## Data Availability

Data is unavailable due to privacy and ethical restrictions; however, it can be provided upon request from the authors.
